# Supplementary material for: An In Silico Molecular Modelling-Based Prediction of Potential Keap1 Inhibitors from Hemidesmus indicus (L.) R.Br. against Oxidative-Stress-Induced Diseases
Source: Molecules. 2023 Jun 3;28(11):4541. doi: 10.3390/molecules28114541 (PMC10254626; doi:10.3390/molecules28114541)

Supplementary Table S1. List of bonding interactions between selected three bioactive compounds and standard drug CPUY192018 with Keap1 protein.

Compound (CID)	Residues	Amino acid	Distance (Å)	Bond category
Hemidescine 101664025	366X	ALA	3.77	Hydrophobic
	514X	VAL	3.85	Hydrophobic
	561X	VAL	3.80	Hydrophobic
	367X	GLY	2.89	Hydrogen
	415X	ARG	2.67	Hydrogen
	416X	ILE	2.32	Hydrogen
	418X	VAL	2.76	Hydrogen
	561X	VAL	3.26	Hydrogen
	561X	VAL	2.68	Hydrogen
	563X	GLN	3.33	Hydrogen
	326X	ARG	4.85	Salt bridge
Beta-Amyrin 73145	366X	ALA	3.90	Hydrophobic
	418X	VAL	3.44	Hydrophobic
	514X	VAL	3.51	Hydrophobic
	561X	VAL	3.93	Hydrophobic
Quercetin 5280343	366X	ALA	3.93	Hydrophobic
	367X	GLY	2.14	Hydrogen
	415X	ARG	1.89	Hydrogen
	415X	ARG	2.30	Hydrogen
	465X	VAL	2.31	Hydrogen
	465X	VAL	2.44	Hydrogen
	510X	ALA	2.08	Hydrogen
	512X	VAL	2.72	Hydrogen
	606X	VAL	2.39	Hydrogen
CPUY192018 73330369	514X	VAL	3.66	Hydrophobic
	367X	GLY	2.46	Hydrogen
	418X	VAL	1.55	Hydrogen
	465X	VAL	1.97	Hydrogen
	561X	VAL	2.09	Hydrogen
	608X	VAL	3.26	Hydrogen

Supplementary Figure S1: Keap1 protein grid box

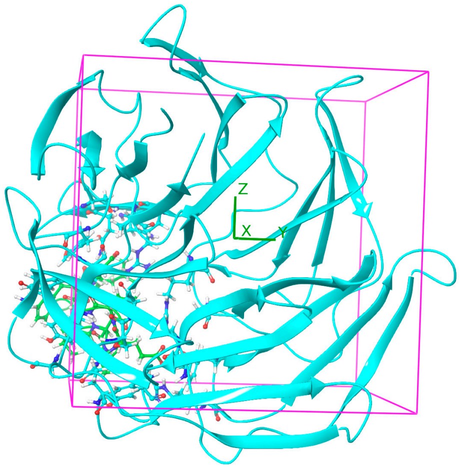

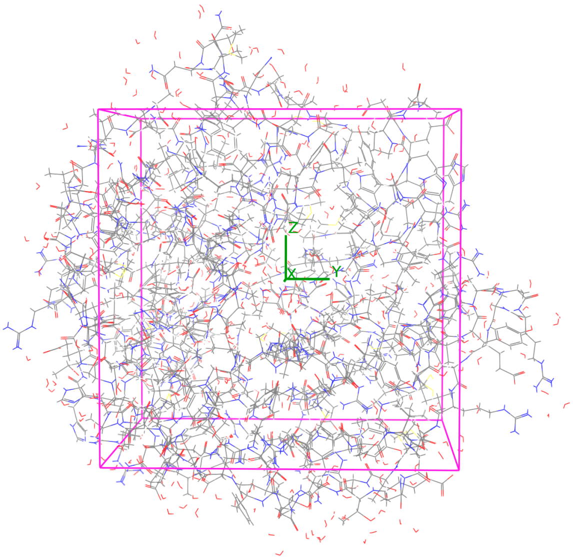

Supplement: Supplementary file 1 [file molecules-28-04541-s001.zip › molecules-2408314-supplementary.pdf]
